# Cisternal Blood Clearance Attenuates Systemic Inflammatory Response After Subarachnoid Hemorrhage

**DOI:** 10.1007/s12028-024-02112-0

**Published:** 2024-10-29

**Authors:** Marco Bissolo, Istvan Csók, Christian Scheiwe, Jürgen Grauvogel, Jürgen Beck, Eva Rohr, Klaus-Jürgen Buttler, Peter C. Reinacher, Roland Roelz

**Affiliations:** 1 Department of Neurosurgery, Medical Center - University of Freiburg, Faculty of Medicine, University of Freiburg, Breisacher Str. 64, 79106 Freiburg, Germany; 2 Department of Stereotactic and Functional Neurosurgery, Medical Center - University of Freiburg, Faculty of Medicine, University of Freiburg, Freiburg, Germany; 3 Fraunhofer Institute for Laser Technology (ILT), Steinbachstraße 15, 52074 Aachen, Germany

**Keywords:** Cisternal blood clearance, Systemic inflammatory response, Subarachnoid hemorrhage, Systemic inflammatory response syndrome

## Abstract

**Background:**

Aneurysmal subarachnoid hemorrhage (SAH) frequently triggers systemic inflammatory response syndrome (SIRS). SIRS has been associated with inferior outcomes and, specifically, delayed cerebral infarction after aneurysmal SAH. Here, we assess the impact of intracranial blood clearance through stereotactic catheter ventriculocisternostomy on SIRS in patients with aneurysmal SAH.

**Methods:**

We assessed daily SIRS criteria (heart rate > 90 beats/min, respiratory rate > 20 breaths/min or abnormal respiratory coefficient, temperature > 38 °C or < 36 °C, white blood cell count < 4000 or > 12,000 cells/mm^3^) between admission and day 21 in 80 consecutive patients who underwent cisternal lavage through stereotactic catheter ventriculocisternostomy from 2015 to 2022. These patients were compared with 80 matched controls who received treatment at our institution between 2010 and 2015. We conducted a mixed effects model analysis using restricted maximum likelihood estimation to assess the effects of treatment groups on the SIRS rate while accounting for repeated measures. Additionally, Bonferroni’s correction was employed to examine specific differences between groups at different time points.

**Results:**

The mean percentages of patients meeting SIRS criteria during the first 21 days after aneurysmal SAH were 23% in the matched cohort group and 14% in patients who underwent cisternal lavage (*p* < 0.001). Additionally, significant differences were observed in the mean leukocyte count (*p* = 0.047), mean heart rate (*p* = 0.019), and mean respiratory rate (*p* = 0.0018) between the two groups. However, there was no significant difference in mean body temperature (*p* = 0.36).

**Conclusions:**

Intracranial blood clearance and cisternal lavage after aneurysmal SAH is associated with a decline in SIRS prevalence and severity.

## Introduction

Systemic inflammatory response syndrome (SIRS) is a well-known phenomenon in various types of acute cerebral injury [1–4]. The pertinent literature shows that the presence of SIRS is associated with worse outcome in patients with aneurysmal subarachnoid hemorrhage (SAH) [3, 5, 6]. Moreover, a higher burden of SIRS in the initial 4 days independently predicts symptomatic vasospasm [5].

SIRS is defined according to four clinical and laboratory parameters: body temperature, heart rate, oxygenation index or respiratory rate, and white blood cell count. SIRS is manifest when two of the following conditions are met: temperature of < 36 °C or > 38 °C, heart rate of > 90 beats/min, respiratory rate of > 20 breaths/min, and white blood cell count of < 4000 or > 12,000 cells/mm^3^ [3, 5, 6]. These clinical signs reflect a systemic process associated with activation of inflammatory factors, endothelial dysfunction, and activation of complement and coagulation cascades with potential thrombosis and impaired microcirculatory flow [5]. This leads to altered tissue perfusion and promotes organic damage, worsening the overall outcome.

Stereotactic catheter ventriculocisternostomy (STX-VCS) and fibrinolytic/spasmolytic lavage is a new method for intracranial blood clearance and, hence, prevention of delayed cerebral infarction (DCI) [6, 7]. We have previously shown in an unselected aneurysmal SAH patient population that the DCI rate and burden can be considerably reduced, and outcomes may be improved with implementation of this treatment (reduction in the overall rate of DCI [18 vs. 7.9%] and total DCI volumes [8933 mL (100%) and 3329 mL (36%)]) [7]. The present study was performed to assess the impact of intracranial blood clearance on SIRS prevalence and severity.

## Methods

### Study Design

This retrospective monocentric study was approved by the independent ethics committee of our medical center (reference no: 575/16). All consecutive patients with aneurysmal SAH treated at the Department of Neurosurgery, Freiburg University Hospital, in a 10-year period between April 2010 and June 2020 were included. Patients with nonaneurysmal SAH, traumatic SAH, early mortality (i.e., within 96 h of admission), and admission delay (patients admitted more than 96 h after the onset of the bleed) were excluded from the study.

Data collection was performed by evaluating electronic patient records, electronic patient charts from the intensive care unit (ICU), and available imaging. The following inclusion criteria were defined: evidence for SAH on cranial computer tomography (CT) or magnetic resonance imaging (MRI); evidence of aneurysm on CT, MRI, or conventional angiography; and presence of at least one follow-up imaging performed more than 24 h after initial imaging.

STX-VCS and intracranial blood clearance through cisternal lavage became available as a novel therapy for the prevention of DCI in 2015. Patients considered at high risk for DCI were offered STX-VCS based on individual treatment decisions. The method and details of STX-VCS and cisternal lavage have been described in previous publications [6, 7]. Briefly, a catheter is implanted transventricularly under stereotactic guidance into the prepontine cistern, creating a catheter ventriculocisternostomy. Once the catheter is placed, cisternal lavage with electrolyte solution containing 100 IU/mL of urokinase begins. Should vasospasm occur during the ICU course, fibrinolytic lavage therapy will be replaced by nimodipine for vasodilation. The external ventricular drain is used as irrigation outflow tract. An intracranial pressure probe is placed for pressure monitoring [6, 7]. This treatment approach has been established as a standard of care for patients with a high risk of developing vasospasm, as assessed through clinical factors such as the Hijdra score, Fisher grade, and World Federation of Neurosurgical Societies grade. Catheter placement was performed usually within 72 h after hemorrhage. For patients initially deemed to have a low risk of developing symptomatic vasospasm but who subsequently did develop vasospasm, we conducted what we refer to as “rescue STX-VCS” implantation after the 72-h period.

Standard intensive care in accordance with current guidelines for aneurysmal SAH treatment was provided without changes throughout the treatment period in both cohorts. In our studies, we defined DCI as radiologically confirmed delayed infarction according to the Vergouwen criteria, that is, new infarctions on CT or MRI 3–6 weeks after aneurysmal SAH that were not present on imaging performed within 24–48 h after early aneurysm securing and excluding infarcts related to medical procedures (i.e., external ventricular drain tracts, etc.) [8].

### Assessments

Daily SIRS criteria (heart rate > 90 beats/min, respiratory rate > 20 breaths/min or respiratory coefficient, temperature > 38 °C or < 36 °C, and white blood cell count < 4000 or > 12,000 cells/mm^3^) between admission and day 21 were assessed by an independent and interdisciplinary rating committee consisting of physicians experienced in the treatment of aneurysmal SAH in patients admitted between April 2010 and June 2020. Furthermore, we assessed patient days with SIRS: the average number of days when two or more of SIRS criteria were positive. To measure a possible correlation between amount of cisterna blood and SIRS, we examined the Hijdra score in all patients. Data collection was performed by evaluating electronic patient records, images, and electronic patient charts from the ICU as described in the Study design section.

### Matched Pairs Analysis

A matched pairs analysis was performed to compare patients selected for STX-VCS with appropriate controls. Each patient treated with STX-VCS was matched with a control patient. Criteria for the matching process were defined as follows, with patients being matched on all criteria: sex and age, World Federation of Neurosurgical Societies grade, aneurysm location, method of aneurysm treatment (coiling, clipping), presence or absence of parenchymal hemorrhage, presence of hydrocephalus, Hijdra score and modified Fisher grade, Charlson comorbidity index, and presence of signs of brainstem herniation.

All patients in the matched pairs analysis were initially ventilated, which makes the respiratory rate alone less indicative. Therefore, we incorporated the respiratory coefficient as an additional parameter to account for the mechanical ventilation status of our patient cohort. Specifically, the respiratory coefficient in ventilated patients gives a very precise status of the pulmonary function. This allowed us to consider a broader range of factors and ensure that our SIRS criteria were applicable and accurate in the context of our study population.

### Statistical Analysis

Statistical analyses were performed using data from 528 patients with aneurysmal SAH. Matching of study participants was performed using a stacked approach, and the matching effectiveness was evaluated using a χ^2^ test. Normally distributed patient characteristics were presented as means with standard deviation, nonnormally distributed characteristics were presented as medians, and categorical characteristics were presented as frequencies. Univariate statistical analysis was performed using the statistical program Wizard version 1.9.42(267). The Mann–Whitney *U*-test was used to compare continuous variables without normal distribution. Fisher’s exact test or Pearson’s χ^2^ test was used to analyze categorical variables.

We conducted a mixed effects model analysis using restricted maximum likelihood estimation to assess the effects of treatment groups on the SIRS rate while accounting for repeated measures. Additionally, Bonferroni’s correction was employed to examine specific differences between groups at different time points. The mixed effects model analysis was performed using GraphPad Prism (version 8.4).

## Results

### Baseline Characteristics

A total of 528 consecutive patients with aneurysmal SAH admitted to our center over a 10-year period (April 2010 to June 2020) were included in this study. Two hundred eighty-seven patients were admitted in the 5-year period immediately before STX-VCS became available. STX-VCS was performed in the second half. Eighty of 241 patients (33%) were offered STX-VCS on the basis of individual treatment decisions after 2015. There was no difference in the medication regimen during the ICU stay. The team of senior physicians was also the same in both cohorts. The 80 patients who were treated by STX-VCS were matched to an appropriate control cohort from the 287 patients treated before STX-VCS was introduced (before 2015). Details of the patients’ characteristics and their distribution between both groups are shown in Table 1. The characteristics of both groups after matching were largely balanced, but patients treated with STX-VCS featured a significantly higher Charlson comorbidity index.

**Table 1:** Baseline characteristics of the matched pair groups

	Matched pairs analysis		
	Matched controls	STX-VCS	*p* value
Patient number	80	80	
Patient characteristics			
Female, *n* (%)	58 (73)	56 (70)	0.73
Age at diagnosis, median (IQR), years	54 (48–61)	59 (49–64)	0.11
Charlson comorbidity index, median (IQR)	1 (0–2)	2 (1–3)	0.005
DCI, *n* (%)	12 (15)	6 (7.5)	0.13
aSAH characteristics			
Admission WFNS grade, n (%)			0.08
1	1 (1.25)	7 (8.75)	
2	6 (7.5)	2 (2.5)	
3	3 (3.75)	7 (8.75)	
4	15 (18.75)	13 (16.25)	
5	55 (68.75)	51 (63.75)	
Hijdra score, median (IQR)			
Total	26 (19–31)	26 (18–32)	0.46
Ventricles	2 (4–8)	2 (4–10)	0.63
Cisterns	22 (15–27)	21 (12–26)	0.32
Intracerebral hemorrhage, *n* (%)	25 (31.25)	37 (46.25)	0.05
Location of ruptured aneurysms, *n* (%)			0.99
ICA	11 (13.75)	11 (13.75)	
MCA	22 (27.5)	22 (27.5)	
ACA	35 (43.75)	34 (42.5)	
PCA	12 (15)	13 (16.25)	
Aneurysm size mean (± SD), mm	6.1 (± 3.6)	6.1 (± 4.2)	0.99
Aneurysm treatment, *n* (%)		0.49	0.12
Clip	43 (53.75)	33 (41.25)	
Coil	37 (46.25)	47 (58.75)	

ACA, Anterior cerebral artery; aSAH, aneurysmal subarachnoid hemorrhage; DCI, delayed cerebral infarction; ICA, internal carotid artery; IQR, interquartile range; MCA, middle cerebral artery; PCA, posterior cerebral artery; STX-VCS, stereotactic catheter ventriculocisternostomy; WFNS, World Federation of Neurosurgical Societies

**Fig. 1:**
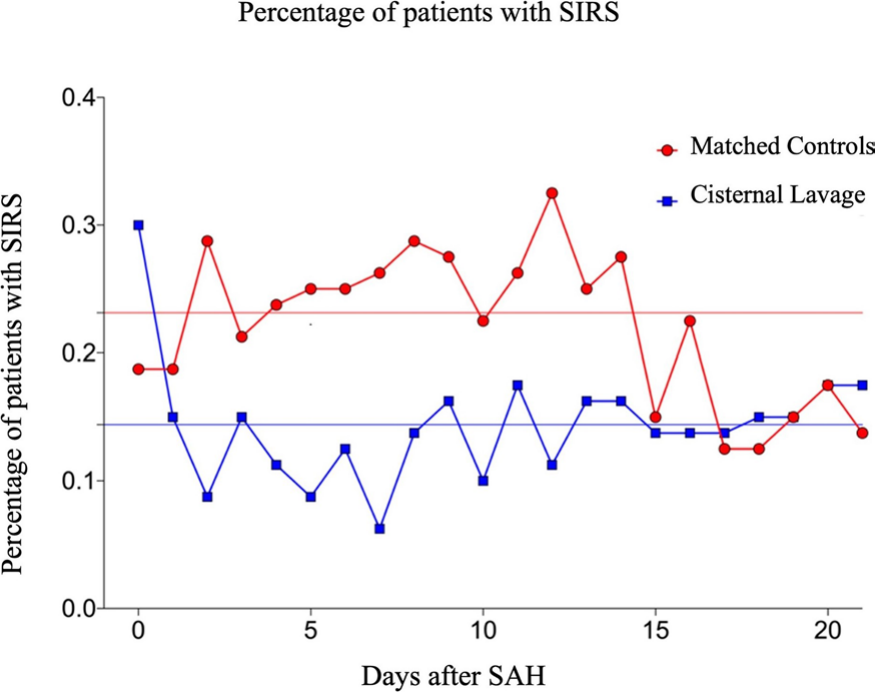
The mean percentage of patients with SIRS criteria on the first 21 days after aneurysmal SAH was 23% in the matched control cohort and 14% in patients treated with STX-VCS (*p* < 0.001). SAH, subarachnoid hemorrhage; SIRS, systemic inflammatory response syndrome; STX-VCS, stereotactic catheter ventriculocisternostomy

Our primary mixed effects model analysis revealed a significant difference between the matched controls and cisternal lavage groups for the rate of SIRS over the first 21 days after aneurysmal SAH, indicating overall group disparities. The mean rates of SIRS were 18.6% in the matched controls group and 9.4% in the cisternal lavage group (*p* > 0.043) (Fig. 1). Further examination through Bonferroni’s correction uncovered specific time points when the difference between the groups was particularly notable. Significant differences were observed on days 2, 4, 5, 6, 7, and 12, with adjusted *p* values of 0.022, 0.024, 0.045, 0.039, 0.013, and 0.019, respectively. Notably, the reduction in SIRS rate started between days 2 and 3 after hemorrhage, namely after implantation of the cisternal catheter and the beginning of the cisternal lavage with urokinase.

**Fig. 2:**
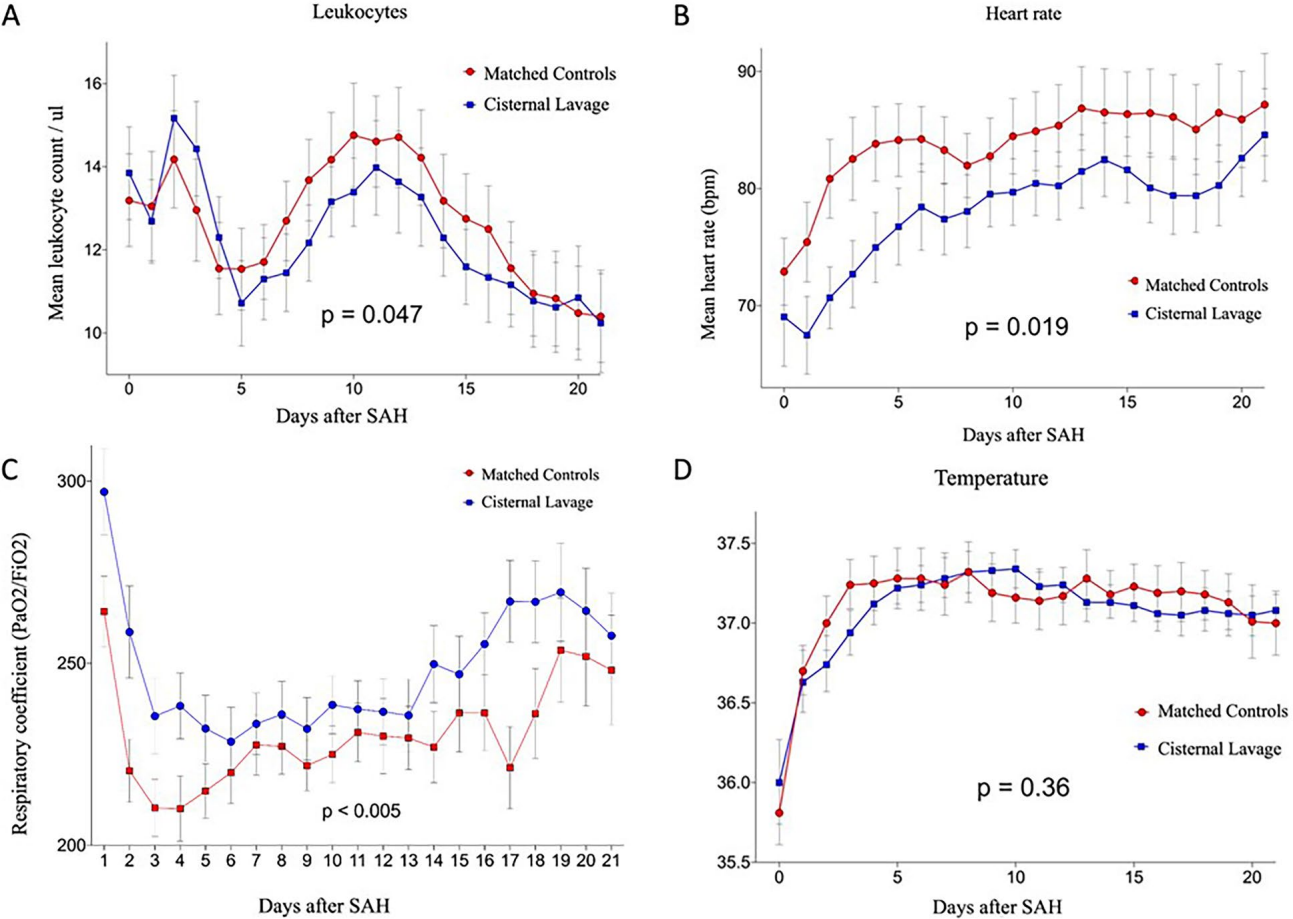
Presentation of individual SIRS parameters in the matched control cohort and patients with STX-VCS on the first 21 days after aneurysmal SAH. Significant differences were found for the mean leukocyte count (**A**) (*p* = 0.047), the mean heart rate (**B**) (*p* = 0.019), and the mean respiratory rate (**C**) (*p* = 0.0018). The mean body temperature was not different between both groups (**D**) (*p* = 0.36). FiO_2_, fraction of inspired oxygen; PaO_2_, partial pressure of arterial oxygen; SAH, subarachnoid hemorrhage; SIRS, systemic inflammatory response syndrome; STX-VCS, stereotactic catheter ventriculocisternostomy

Analyzing the leukocytes count during the first 21 days after SAH, we found significantly lower levels in patients treated with STX-VCS compared with matched controls (*p* = 0.047) (Fig. 2A). Again, the greatest difference between the two groups was found between days 5 and 15, that is, in the period at highest risk of DCI. Patients treated with STX-VCS featured significantly lower average heart rates (*p* = 0.019) (Fig. 2B). In contrast to the leukocyte count, the difference between the two groups was not particularly marked during days 5–15 but rather stable during the observational period. Patients treated with STX-VCS showed a significantly lower respiratory rate compared with the matched control group (*p* = 0.0018). Similarly, the oxygenation ratio was higher in patients who received cisternal irrigation (Fig. 2C). No significant difference was found in the daily mean body temperatures between patients treated with STX-VCS and matched controls (Fig. 2D).

## Discussion

Systemic complications are frequent and represent important contributors to morbidity and mortality in patients with aneurysmal SAH [4]. In addition to secondary neurological injuries, such as DCI, SAH has been associated with nonneurological systemic medical complications, such as SIRS [3, 4].

Yoshimoto et al. [3] demonstrated a clear relationship between SIRS criteria and patients’ short-term outcomes. Three of the four SIRS criteria (heart rate, respiratory rate, and white blood cell count) were significant predictors of outcome per se [3]. Furthermore, multivariate analysis showed that the presence of SIRS is an independent factor for unfavorable outcome even after adjusting for the effects of other prognostic markers, such as the Hunt and Hess scale, Fisher grade, and age [3]. Cytokine-mediated tissue damage is thought to promote mechanisms that lead to clinical worsening due to SIRS [3, 9]. It is hypothesized that mechanisms of central dysregulation secondary to intracranial aneurysm rupture contribute to the development and progression of extracerebral organ dysfunction. According to Yoshimoto et al. [3], 77% of extracerebral organ dysfunction occurred in conjunction with SIRS. More recent data have also described an association between systemic inflammation and poor long-term functional outcomes in aneurysmal SAH [10].

Because of the complex nature of this pathology, it is difficult to understand mechanistically how exactly the inflammatory cascade begins. Blood products and damage-associated molecules released from injured or stressed peripheral and central nervous system cells after the initial insult can potentially initiate the inflammatory cascade both in the brain parenchyma and cerebral vessels and in the systemic circulation and link to the delayed phase of post-aneurysmal-SAH complications [3, 4, 11].

Cisternal lavage therapy using fibrinolytics to remove subarachnoid blood and its metabolites and spasmolytics to directly target vasospastic vessels represents an alternative approach to DCI prevention [6, 7]. Our department implemented STX-VCS to deliver cisternal lavage into the management of patients with aneurysmal SAH in 2015 [6, 7]. Topical effects on the brain, such as reduction of cerebral vasospasm or DCI has already been shown in recent studies [7]. Intrathecal fibrinolysis appears to reduce spasmogen levels by delivering fibrinolytic agents into the subarachnoid space, removing blood metabolites and proinflammatory molecules, and restoring normal cerebrospinal fluid [7]. Furthermore, cisternal therapy directed at blood clearance seems to be more effective for the prevention of cerebral vasospasm and delayed infarction by removing damage-associated molecules compared with cisternal rescue spasmolysis [12].

Our analysis revealed a significant difference between the matched controls and the STX-VCS group for the rate of SIRS over the first 21 days after aneurysmal SAH, indicating overall group disparities. The mean rates of SIRS were 18.6% in the matched controls group and 9.4% in the STX-VCS group, even though every patient treated with STX-VCS needed an additional surgery for catheter implantation. The relationship between surgical stress and inflammatory response is well known but has not been well described in the neurosurgical population. The literature is controversial on the differential risk between aneurysm treatment modalities. Diringer et al. [13] support an inflammatory mechanism through which surgery might contribute to adverse outcomes.

The decline of SIRS prevalence was strongest in the period between days 5 and 15, the period of maximum DCI risk. Interestingly, the decrease in SIRS rate began between the second and third day after hemorrhage, that is, after implantation of the cisternal catheter and initiation of cisternal lavage with urokinase. Notably, Dhar and Diringer [5] found that SIRS occurring in this phase was of highest relevance for the subsequent development of cerebral vasospasm.

No difference, however, was found from day 15, when both cohorts showed comparable levels of SIRS. However, this was due to a decrease in the risk of SIRS in the matched control cohort rather than an increase in the STX-VCS cohort. Interestingly, we found a significant decrease in three of four SIRS parameters, namely white blood cell count, respiratory rate/oxygenation index, and heart rate. No difference was found in the mean temperature between the matched control group and patients treated with STX-VCS. The temperature disturbance in patients with aneurysmal SAH has been partly attributed to an increased burden of SAH and intraventricular hemorrhage, possibly due to a change in temperature set point due to toxic and inflammatory effects and/or ischemic complications in the brainstem/hypothalamus. Potentially, this set point change is due to the initial hemorrhage and is not influenced by cisternal lavage.

Our study is subject to the general constraints of retrospective analyses. Primarily, causal relationships require randomized investigations and cannot be established on the basis of retrospective data. However, the study is useful for providing preliminary data and in guiding the development of future prospective studies. We initiated a randomized trial to assess safety and efficacy of STX-VCS (EudraCT identifier: 2017-000868-15). Assessment of adverse events in this trial will be performed to test the observations reported here.

We tried to exclude bias on the study end points by independent and blinded assessments wherever possible and reporting of an unselected patient series. In particular, extraction of SIRS criteria as a central element of the study was performed by a physician who was not involved in the treatment of the patients and blinded for the study hypothesis. Time is a principal limitation of retrospective cohort studies. However, no relevant changes in patient management other than STX-VCS have been made during the period concerned in our department. In this analysis, we simply defined SIRS as present or absent, which does not capture the continuous spectrum of systemic inflammation after SAH. Although data were matched, we cannot exclude that unmeasured confounders could have affected the results. SIRS criteria, although extremely sensitive, lack specificity. Because differentiation between SIRS and sepsis was not always possible given the retrospective nature of the study, we decided not to include this differentiation in our analysis. Because of the consistency of the ICU therapy, we believe that the lack of this differentiation would not affect the results of study.

## Conclusions

Active blood clearance after an aneurysmal SAH through cisternal lavage not only directly affects cerebral vessels by reducing vasospasm and DCI but also impacts the systemic effects of the hemorrhage. Cisternal lavage is linked to a decrease in both the prevalence and severity of SIRS in patients with SAH.
